# Textile Concentric Ring Electrodes for ECG Recording Based on Screen-Printing Technology

**DOI:** 10.3390/s18010300

**Published:** 2018-01-21

**Authors:** José Vicente Lidón-Roger, Gema Prats-Boluda, Yiyao Ye-Lin, Javier Garcia-Casado, Eduardo Garcia-Breijo

**Affiliations:** 1 Instituto Interuniversitario de Investigación de Reconocimiento Molecular y Desarrollo Tecnológico (IDM), Universitat Politècnica de València, Universitat de València, Valencia 46022, Spain; jvlidon@eln.upv.es; 2 Centro de Investigación e Innovación en Bioingeniería, Universitat Politècnica de València, Valencia 46022, Spain; gprats@ci2b.upv.es (G.P.B.); yiye@eln.upv.es (Y.Y.L.); jgarciac@ci2b.upv.es (J.G.C.)

**Keywords:** textile electrode, concentric ring electrode (CRE), Laplacian electrocardiogram, PEDOT:PSS

## Abstract

Among many of the electrode designs used in electrocardiography (ECG), concentric ring electrodes (CREs) are one of the most promising due to their enhanced spatial resolution. Their development has undergone a great push due to their use in recent years; however, they are not yet widely used in clinical practice. CRE implementation in textiles will lead to a low cost, flexible, comfortable, and robust electrode capable of detecting high spatial resolution ECG signals. A textile CRE set has been designed and developed using screen-printing technology. This is a mature technology in the textile industry and, therefore, does not require heavy investments. Inks employed as conductive elements have been silver and a conducting polymer (poly (3,4-ethylenedioxythiophene) polystyrene sulfonate; PEDOT:PSS). Conducting polymers have biocompatibility advantages, they can be used with flexible substrates, and they are available for several printing technologies. CREs implemented with both inks have been compared by analyzing their electric features and their performance in detecting ECG signals. The results reveal that silver CREs present a higher average thickness and slightly lower skin-electrode impedance than PEDOT:PSS CREs. As for ECG recordings with subjects at rest, both CREs allowed the uptake of bipolar concentric ECG signals (BC-ECG) with signal-to-noise ratios similar to that of conventional ECG recordings. Regarding the saturation and alterations of ECGs captured with textile CREs caused by intentional subject movements, silver CREs presented a more stable response (fewer saturations and alterations) than those of PEDOT:PSS. Moreover, BC-ECG signals provided higher spatial resolution compared to conventional ECG. This improved spatial resolution was manifested in the identification of P1 and P2 waves of atrial activity in most of the BC-ECG signals. It can be concluded that textile silver CREs are more suitable than those of PEDOT:PSS for obtaining BC-ECG records. These developed textile electrodes bring the use of CREs closer to the clinical environment.

## 1. Introduction

The recording of electrophysiological signals in its simplest form—that is, through contact electrodes attached to the skin—is subject to continuous studies both to optimize these records and in the search for new technologies that improve the measurement process. Today the diagnosis, therapy, and monitoring of health are based to a large extent on the measurement of signals from the brain, heart, and muscles. Even so, most of the recording systems of these signals continue to have a traditional approach, using monopolar disk electrodes (mainly Ag or AgCl). In recent years, an effort has been made to look for alternative geometries and new technologies for the manufacture of contact electrodes that allow signals to be obtained that are of better quality and/or have more precise information. In addition, systems with multi-electrodes that allow the recording of several signals simultaneously are being imposed. Finally, the integration of electrodes into clothes is being sought, which would lead to medical control beyond the clinical work environment. The use of textile-based electrodes entails a series of special characteristics such as ultra-thinness, light-weighted, high flexibility, stretchability, and conformity [1]. There are two tendencies for the realization of generic textile-based electrodes: printing the electrodes on the textile using different types of inks and printing techniques [2,3,4,5,6] or using fibers and weaving or sewing the electrodes [7,8,9,10]. In particular, on the use of textile-based electrodes for the control of health, there are several very interesting reviews in the literature on this subject [1,11,12,13] that confirm the trends mentioned above.

Two important aspects to consider when designing and using electrodes for measuring bioelectric signals are the materials to be used and where to place them. As for the material, you can find electrodes that are of metal inks, conductive polymer inks or are directly of conductive textile. Each of these materials must provide flexibility to improve contact during movement of the individual. Based on this flexibility, designs can be found with conductive foam [14], conducting polymers (poly (3,4-ethylenedioxythiophene) polystyrene sulfonate (PEDOT:PSS)) and polymers with conductive particles (silver) [15], nanoparticles [16], or carbon nanotubes [17]. As for the location of the electrodes, there are works published in the literature with electrodes arranged in different positions on T-shirts, vests, girdles, and swimsuits [18,19,20].

As mentioned, one of the techniques for manufacturing electrodes for capturing bioelectric signals is direct printing on a substrate. In recent years, works have been developing printing techniques based on graphic arts such as screen printing, gravure, or inkjet for the manufacture of these electrodes on flexible substrates and more specifically on textiles [21,22]. The screen-printing technology is the most used and mature printing technology and has been used for decades in the manufacture of electronic systems. The revolution in the use of screen-printing techniques on flexible substrates occurred with the development of polymer-based inks, which allow low curing temperatures compatible with textile substrates [2].

On the other hand, one of the main limitations of surface bioelectric recording by means of conventional disc electrodes is the poor spatial resolution, mainly originating from the blurring effect due to different conductivities of the body volume conductor [23,24]. To overcome this limitation, surface Laplacian potential records have been proposed [25]. Literature has confirmed that Laplacian records are able to mitigate this effect and provide enhanced spatial resolution surface potential recordings—i.e., they are able to improve the detection of the bioelectric dipole sources closest to the recording electrodes, rejecting the contribution of distant bioelectric dipole sources—when compared to bipolar records made with disk electrodes [24]. First, surface Laplacian potentials were estimated using monopolar disc electrodes and applying discretization techniques [26,27,28]. Subsequently, body surface Laplacian potentials, such as Laplacian electrocardiography (LECG), were obtained by designing and implementing concentric ring electrodes in several configurations (bipolar, quasi-bipolar, and tripolar). Concentric ring electrodes were initially implemented in rigid substrates, mainly printed circuit boards (PCBs) [29]. In that context, Besio et al. [30] developed concentric ring electrodes on PCBs to compare the uptake capacity and spatial sensitivity of different electrode configurations—bipolar conventional (disc electrodes), bipolar concentric, and tripolar—when recording surface electrocardiographic signals. Once the capacity of the ring electrodes to detect the electrocardiographic signal and its higher spatial resolution with respect to the bipolar registers with conventional monopolar electrodes had been demonstrated, Garcia-Breijo et al. [2] compared different printing technologies to make the concentric ring electrodes on flexible plastic substrates (serigraphy, inject-printing, gravure). They concluded that the electrodes with higher reproducibility and better properties for surface bioelectric recordings were those implemented by serigraphy. Then, ring electrodes were developed on flexible plastic substrates with the aim of determining the best dimensions and CRE location to pick up electrocardiographic activity [31,32,33]. Flexible CREs were also developed on plastic substrates to detect uterine electrical activity [34]. Other research groups have also developed flexible electrodes on plastic substrates for capturing different bioelectrical records such as those in electrocardiography (ECG), electroencephalography (EEG) and, to a minor extent, electromyography (EMG) [35,36,37].

Despite the improvements introduced by the implementation of the CRE on flexible substrates, CRE use has not been transferred to the clinical environment. In order to facilitate this, we have worked on the design and validation of a set of two CREs developed on a textile substrate that will improve patient comfort during recording, especially for long-term recordings, and that will enable the detection of bioelectric signals with a similar quality to that of conventional bipolar recordings and with enhanced spatial resolution. In the present work, a set of two concentric ring electrodes screen printed onto a textile substrate has been designed and their features have been compared (electrical characteristics and bioelectric signals quality). Two different types of inks were used: one based on silver and another on a conducting polymer (poly (3,4-ethylenedioxythiophene) polystyrene sulfonate, PEDOT:PSS).

This study is structured in the following way: Section 2 includes the material and methods, describing the design of the CREs, the manufacturing processes, their characterization, the ECG recording protocol and parameters to assess signals quality; Section 3 presents the results corresponding to the CREs’ characterization, ECG signals recorded, and CRE performance; in Section 4 the results are discussed and finally a conclusion is presented in Section 5.

## 2. Materials and Methods 

### 2.1. Textile Concentric Ring Electrodes (CRE): Design and Development

**Figure 1:**
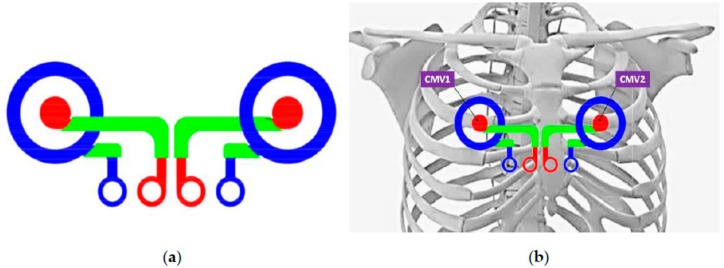
(**a**) Graphic representation of a concentric ring electrode (CRE); (**b**) CRE locations coincide as far as possible with the precordial registration positions CMV1 and CMV2.

The sensing part consists of a set of two concentric ring electrodes, each one made up of an inner disc electrode (Figure 1a) and an outer ring. Although the recording areas of the central disc and the outer ring are not equal, CREs will be connected to commercial bioamplifiers (P511, Grass Technologies, Warwick, RI, USA) with input impedances high enough to disregard the imbalance between the impedances of both poles of the CREs. Taking into account that the CRE’s external diameter should be approximately at the distance between the body surface and the bioelectric sources to be recorded [29,38], the ring’s external diameter was set to 5 cm since the distance between the torso surface and the heart is between 3.5 and 5.0 cm [30]. In addition, textile electrodes could provide a higher impedance electrode with respect to the CRE implemented on plastic substrates [32,33], together with the signal recording being under dry conditions (without electrolytic gel), which could hinder their capability of detecting the ECG signal, we decided to increase the recording area of these textile CREs.

The CRE dimensions are shown in Table 1. Furthermore, the distance between the CREs has been set taking into consideration that it is desired to record ECG signals in positions that are as close as possible to the standard recording positions CMV1 (position comparable to precordial V1 near the right atrium) and CMV2 (comparable to precordial V2 near the left atrium); see Figure 1b.

**Figure 2:**
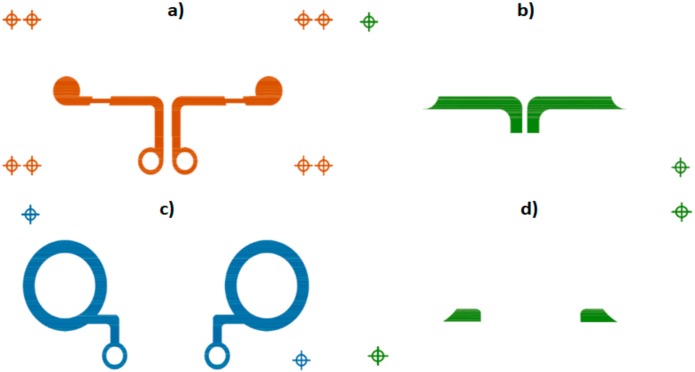
Screen patterns used. (**a**) The first layer corresponds to the disc electrode (conductor layer); (**b**) dielectric that insulates the connection line that joins the inner disc to the connector; (**c**) concentric ring electrode is implemented in the third layer (conductor layer); (**d**) fourth layer, dielectric, insulates the connection line that joins the concentric ring electrode to the connector and the skin.

Manufacturing technology used to implement this type of sensor was based on serigraphic technology of thick film. The screen-printing process consists of forcing inks of different characteristics over a substrate through some screens using squeegees. Openings in the screen define the pattern that will be printed on the substrate by serigraphy. The final thickness of the inks can be adjusted by varying the thickness of the screens. Specifically, textile CREs were produced by screen-printing technology using a four-layer design as shown in Figure 2. The first layer corresponds to the disc electrode (conductor layer). The second layer insulates the connection line that joins the inner disc to the connector, preventing a short circuit with the ring electrode and preserving the shape of the disc electrode. The concentric ring electrode is implemented in the third layer (conductor layer). The fourth layer, similar to the second layer, insulates the connection line that joins the concentric ring to the connector and the skin.

The screen for the conductors was a 230 mesh of polyester material (PET 1500 90/230-48, Sefar, Thal, Switzerland) and the screen for the dielectric layer was a 175 mesh polyester material (PET 1500 68/175-64 PW, Sefar). In order to transfer the stencil to the screen mesh, a UV film Dirasol 132 (Fujifilm, Tokyo, Japan) was used. The final screen thickness was 10 µm for the screen for conductors and 15 µm for the screen for the dielectric. The patterns were transferred to the screen by using a UV light source unit. The materials used were the textile Mediatex TT ACQ 120 µm (Junkers&Muellers gmbh, Mönchengladbach, Germany) for the substrate, C2131014D3 Silver ink 59.75% (Gwent Group, Pontypool, UK) and C2100629D1 PEDOT:PSS (Gwent Group, Pontypool, UK) as the conductive inks and D2081009D6 polymer dielectric (Gwent Group, Pontypool, UK) as the dielectric ink. Flexibility is one of the most important characteristics of these inks in order to use them with textiles. Their main characteristics are shown in Table 2. Sheet resistivity (Ω/sq) measured was 76 mΩ/sq for Ag and 268 Ω/sq in the case of PEDOT:PSS for final thickness obtained. Printing was carried out using an Ekra E2 XL screen-printer (ASYS Group GmbH, Dornstadt, Germany) with a 750 shore squeegee hardness, 3.5 bar force, and 8 mm/s. After depositing the inks, they were cured in an air oven (UNB-100 Memmert GmbH+Co.KG, Schwabach, Germany) at 130 °C for 3 min (Ag ink) and 15 min (PEDOT:PSS ink).

**Figure 3:**
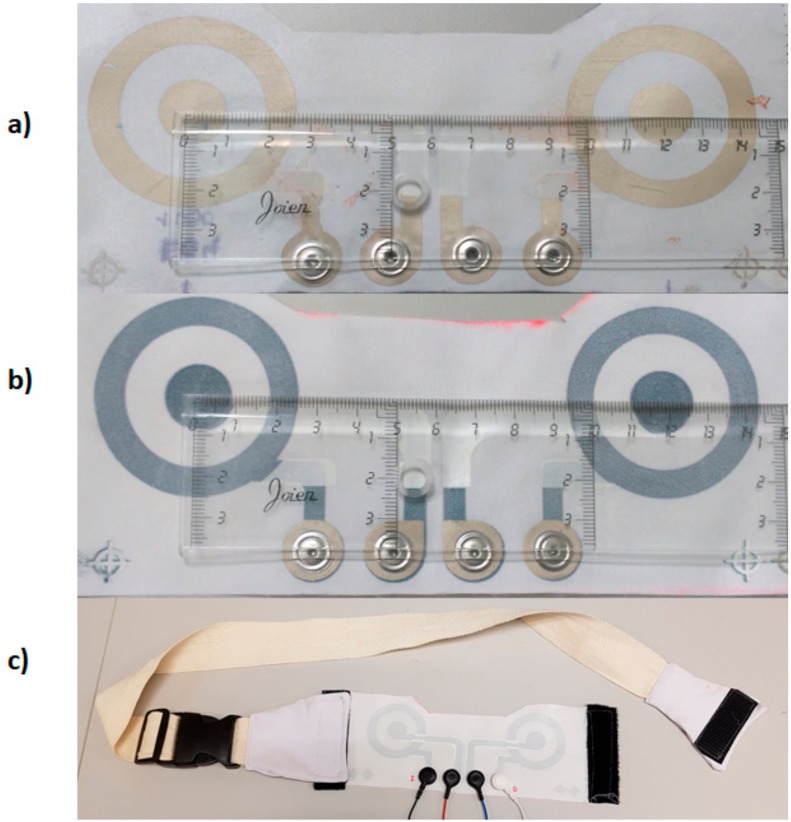
Photograph of the CRE set implemented: (**a**) corresponds to the silver electrode, (**b**) corresponds to the PEDOT:PSS (poly (3,4-ethylenedioxythiophene) polystyrene sulfonate) electrode and (**c**) CRE integrated with an adjustable belt.

Figure 3 is a photograph of the CREs implemented with silver (Figure 3a) and with PEDOT:PSS (Figure 3b) inks. To facilitate the electrical connection with the measuring system, a snap (Sparkfun) was incorporated at each terminal of the electrodes as shown. Due to the relationship between the electrode-skin contact and signal quality, in this first prototype, the textile CREs were integrated into an adjustable belt (see Figure 3c) that exerted a certain pressure on the chest contour so as to guarantee the electrode-skin contact. In this sense, it is well known that as pressure on the chest contour increases, the skin-electrode impedance is reduced and, therefore, a better signal quality is obtained [39,40]. This belt is to be placed in a supramamarian position, making the electrodes coincide as far as possible with the registration positions CMV1 and CMV2.

### 2.2. Physical and Electrical Electrode Characterization 

A physical characterization was made by measuring the final layer thickness with Profilm3D (Filmetrics) with a 20× Mirau objective.

An electrical characterization was carried out by measuring the magnitude of the impedance and the angle of the phase by using electrochemical impedance spectroscopy, which was taken with a potentiostat (Bio-Logic SP-300, Bio-Logic Science Instruments, Seyssinet-Pariset, France) in a two-electrode configuration supplying a sinusoidal signal of 1 V without any dc bias. The measurement was made between the two terminals of one of the electrodes (pole-to-pole impedance), both with skin contact.

The skin-electrode impedance of each CRE pole was carried out using the EIM-105 Prep-Check (General Devices Co Inc., Indianapolis, IN, USA) in a three-electrode configuration at 10 Hz.

### 2.3. Recording Protocol

In total, ten recording sessions were performed with 7 male and 3 female healthy subjects aged between 20 and 41 years and with body mass indices between 19.6 and 27.4 kg/m^2^. The recordings were carried out with the subjects lying on a stretcher. This study was approved by the Polytechnic University of Valencia Ethics Committee and adhered to the Declaration of Helsinki. The volunteers were informed about the nature of the study and briefed on the recording protocol before they signed a consent form.

**Figure 4:**
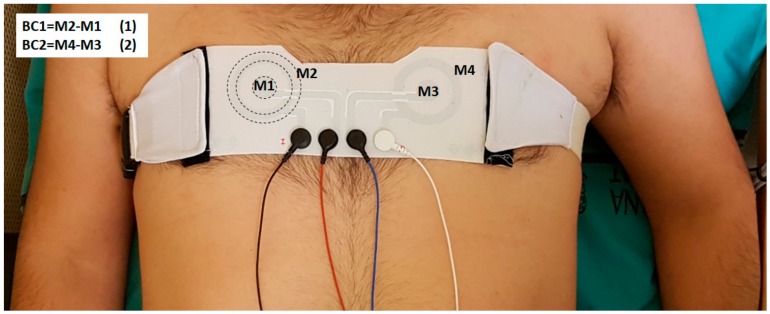
Attachment of the concentric ring electrode to the chest for obtaining two BC-ECG recordings simultaneously. M1: inner disc of the patient's right electrode, M2: outer ring of the patient’s right electrode, M3: inner disk of the patient’s left electrode and M4: outer ring of the patient's left electrode.

To reduce contact impedance, the skin area on which the electrodes (conventional and concentric) were placed was previously gently exfoliated (Nuprep, Weaver and Company, Aurora, IL, USA) and was also shaved in the case of subjects with excess hair. Two disposable Ag/AgCl electrodes (Kendall 100 series Foam electrodes, Medtronic, Minneapolis, MN, USA) were positioned on the left leg and right arm for obtaining standard lead-II ECG signals with the ground electrode being on the right leg. First, the silver CRE, previously cleaned with alcohol, was attached to the chest with an adjustable belt that exerted a certain pressure on the skin. The right electrode was positioned as close as possible to CMV1 (position comparable to V1 near to the right atrium) (see Figure 4). Immediately after placing the electrodes, the skin-electrode impedance of each CRE pole was measured as indicated in the previous section. Subsequently, two bipolar concentric ECG (BC-ECG) signals using silver CRE and standard lead-II ECG signals were recorded for 1 minute with the subject at rest. For obtaining and conditioning the two BC-ECG and lead II ECG, commercial instrumentation amplifiers (Grass Technologies P511, AstroNova, Inc., West Warwick, RI, USA) were used. Since the main information of ECG signals is distributed in the bandwidth 0.1–100 Hz [41], signals were band-pass filtered in this bandwidth and then acquired with a sampling rate of 1000 Hz. Subsequently, the electrodes’ sensitivity to possible movements was analyzed. For this purpose, 1-minute of all BC-ECGs and lead II signals were recorded with the subject performing the movements listed for 10 s: lateral head movement, vertical arm movement, vertical leg movement, laughing, and deep breathing. Subsequently, the silver CRE was replaced with the PEDOT electrode and the skin-electrode impedance measurement and ECG signal acquisition during rest and during motion were repeated.

### 2.4. ECG Analysis

ECG can be corrupted by background noise and different types of interferences, such as baseline drift and power line and abdominal muscle interference. First, the ECG signals were digitally filtered with a fifth-order Butterworth high pass filter with the cutoff frequency being at 0.3 Hz. ECG fiducial points were then obtained by detecting the R wave of the ECG signal with the algorithm proposed by Pan and Tompkins [42] and slightly modified by Hamilton and Tompkins [43]. Then, the averaged beat ($\bar{ECG}$) extending from 275 ms prior to the R wave to 425 ms after it was computed.

To compare the ECG signals recorded by the silver and PEDOT:PSS CREs during rest, the peak-peak amplitude of the averaged beat ($\bar{ECG}$) and the signal-to-noise ratio (SNR) were worked out. The latter is defined as the ratio of the root mean square (rms) value of the average beat ($\bar{ECG}$) and that of the noise during the isoelectric period between beats.

The ECG signals sensed during intentional movement was analyzed in order to quantify the motion artifact sensitivity of these electrodes. For this purpose, the time percentage in which the ECG signal presented alterations with respect to the sensed signal during rest and/or signal saturation due to each movement was annotated for the signals sensed in each position (left and right). In the present study, signal alteration was considered as any visually appreciable variation of the ECG signal recorded during movement with respect to that obtained at rest. Alterations consisted mainly of baseline changes. By contrast, signal saturation was referred to as amplified signals that reached the maximum or minimum output voltage allowed for conditioning the system, made up by the commercial P511 bioamplifiers connected to the DAQ NI USB 6229 (National Instruments, Austin, TX, USA) with the saturation voltage being ±5 V. Then, the mean time percentage of all the involved subjects was computed for each movement and each position.

## 3. Results

### 3.1. CREs Physical and Electrical Characteristics

**Figure 5:**
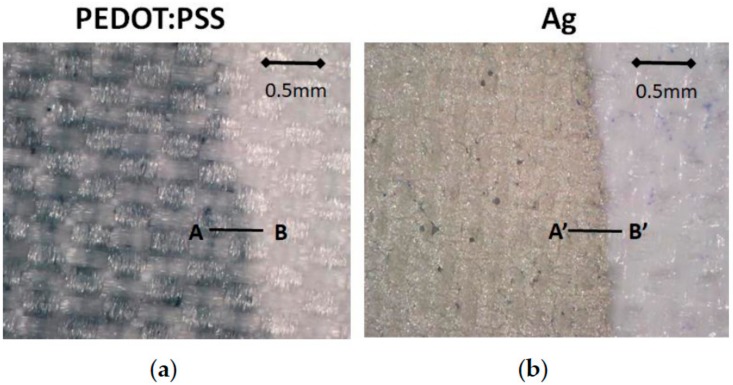
(**a**) Detail of the PEDOT:PSS on the substrate; the PEDOT:PSS is embedded in the fabric pattern; (**b**) Detail of the Ag on the substrate.

**Figure 6:**
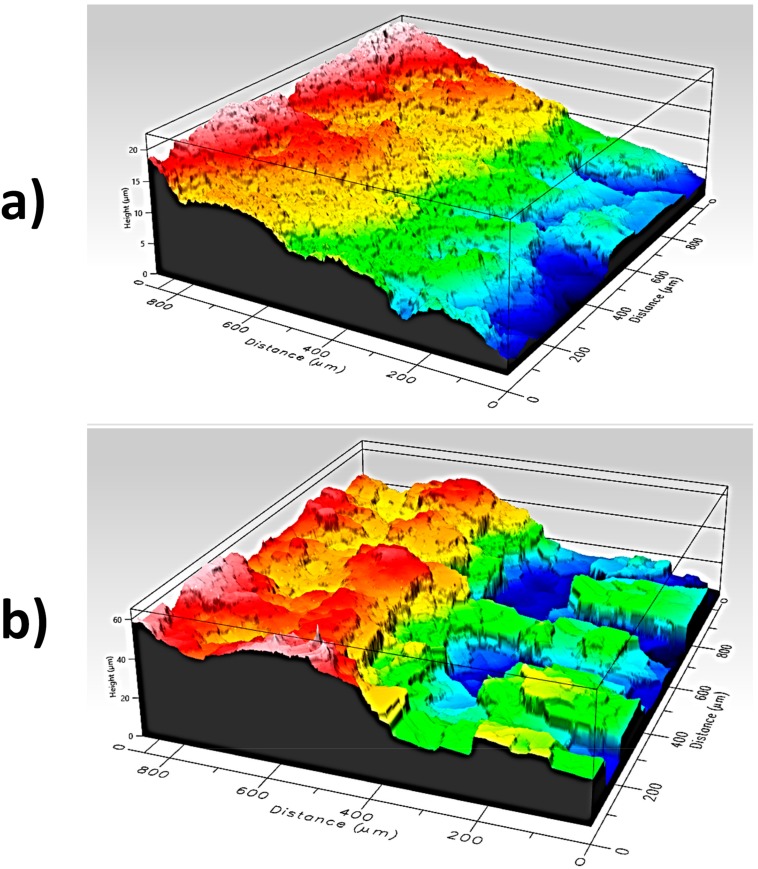
(**a**) Thickness of the PEDOT:PSS on the substrate (view A–B from Figure 5a); (**b**) thickness of the Ag on the substrate (view A’–B’ from Figure 5b).

A magnified view of the two designs is shown in Figure 5 (Figure 5a for PEDOT:PSS and Figure 5b for Ag). As the PEDOT:PSS was embedded in the fabric while the silver remained on the fabric, the different effective thicknesses were obtained in both cases. In the case of PEDOT:PSS, the average thickness obtained was 15 μm (Figure 6a); in the case of the silver, the average thickness obtained was 40 μm (Figure 6b).

**Figure 7:**
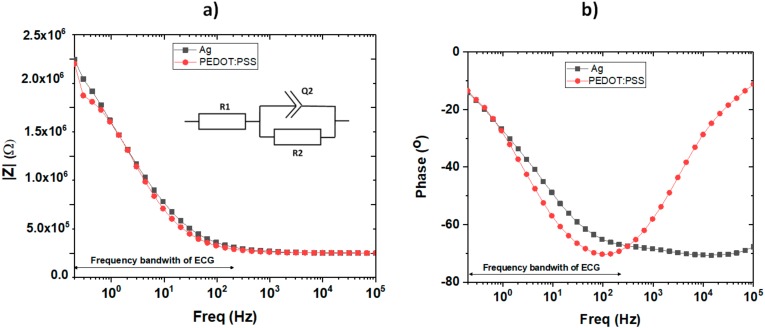
External ring: (**a**) electrode–skin impedance (pole-to-pole) magnitude and (**b**) phase angle.

Regarding the electrical characterization, Figure 7 shows the impedance and phase of the electrodes between 0.1 and 200 Hz—frequency bandwidth of ECG—both in the case of electrode-skin contact.

The electrode impedance characteristics were analyzed using a modified Ershler-Randles equivalent circuit called ZARC—shown in the inset of Figure 7—the equation for which is shown in (3) where R_1_ is the series resistance, R_2_ is the charge-transfer resistance, Q_2_ is the constant phase element, and the exponent α determines the character of frequency dependence. R_1_ represents the resistance between electrodes through the skin, R_2_ represents the transfer of electrons by redox between the electrodes and the skin and Q_2_ can vary by the roughness, thickness, and composition of the material. Table 3 summarizes the data of these variables for the case of using the silver or the PEDOT:PSS.

$$Z\left(f\right)=R_{1}+\frac{R_{1}}{R_{2}\cdot Q_{2}{\left(i\cdot \pi \cdot f\right)}^{\alpha }+1}$$

The behavior of the two electrodes in the working frequency range is very similar. The difference in the value of Q2 makes sense since they are two different materials with different thickness and roughness. It must be taken into account that PEDOT:PSS is permeable to cations and a redox process occurs in its presence.

The skin-electrode impedance measurements during the recording sessions are shown in Table 4. The skin-electrode impedance values presented a high variability among the subjects and were relatively higher than the conventional pre-gelled Ag/AgCl electrodes, which usually provide a skin-electrode impedance lower than 10 kΩ; however, they were still within the admissible limit for bioelectrical signal acquisition. They were also higher than that obtained by the silver CRE implemented on other flexible substrates: Valox, Melinex, Ultem [31]. In comparison to the PEDOT:PSS electrode, the silver electrode impedance was generally lower.

### 3.2. ECG Analysis

**Figure 8:**
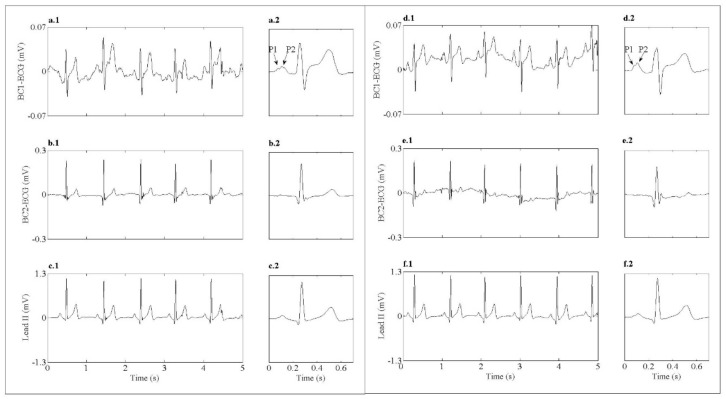
Five seconds of raw ECG signals and its corresponding averaged beat at its right. (**a.1**) BC1-ECG acquired with the silver CRE at the right position (CMV1). (**b.1**) BC2-ECG acquired with the silver CRE at the left position. (**d.1**) BC1-ECG acquired with the PEDOT:PSS CRE in the right position (CMV1). (**e.1**) BC2-ECG acquired with the PEDOT:PSS CRE in the left position. (**c.1**,**f.1**) Standard lead II simultaneously recorded with the two BC-ECG signals sensed by the silver and PEDOT:PSS CREs, respectively. (**a.2**–**f.2**) averaged beats of BC-ECG signals shown in traces (**a.1**–**f.1**) respectively.

Figure 8 shows 5 seconds of the simultaneous recordings of standard lead II (trace c.1 and f.1) and two BC-ECG recordings acquired using the silver electrode (trace a.1 and b.1) and the PEDOT:PSS electrode (trace d.1 and e.1) with the subject at rest. Their corresponding averaged beats are shown on the right side. Initially, fiducial points of ECG signals can be clearly identified in all BC-ECG recordings using both silver and PEDOT:PSS electrodes, being of the BC-ECG signal quality. In addition, the P1 and P2 waves corresponding to the depolarization of the right and left atria can be clearly identified in the BC-ECG averaged beat at the right position (CMV1, trace a.2 and d.2), regardless of the CRE conductor material (silver or PEDOT:PSS). The relative amplitude of the P wave with respect to the QRS complex of BC-ECG acquired at the right position (CMV1) was much higher than that of the standard lead-II ECG signal. By contrast, the signal amplitudes recorded at the left position was higher than that sensed at the right position, although the P wave associated with atrial activity was not appreciated (see traces b and e) since the CRE was positioned away from the atrium. When comparing the BC-ECG signals acquired with the silver and PEDOT:PSS electrodes, no significant morphology change was observed except for the signal amplitude, which may be due to the fact that the silver and PEDOT:PSS electrodes were not positioned exactly in the same position.

Regarding the ECG signals detected during rest, it can be observed in Table 5 that signal amplitude presented a high inter-subject variability with higher ECG signals being detected in the left position. In addition, the amplitude of the signals sensed by the PEDOT:PSS electrode was slightly higher than that obtained by the silver electrode. In contrast, similar signal to noise ratio (SNR) values (around of 21 dB) were obtained for both electrodes regardless of their position.

To quantify motion artifact sensitivity of both CREs, ECG signals were acquired during intentional movements and analyzed. Table 6 shows the mean time percentage in which ECG signals were altered and/or saturated for the total of patients. The last row shows the mean and deviation of the mean time percentage for all of the intentional movements. The mean time percentage of the saturated signal for the silver electrode was lower than 2% regardless of its position. In addition, with respect to the signal sensed during rest, the ECG signal presented alterations almost all of the time in which the intentional movements were generated (~16%). In contrast, using the PEDOT:PSS electrode, the signals remained altered for a longer period (25–30%), even after the movement had finished. These electrodes were in general more sensitive to motion artifact with the mean time percentage of the altered signal and saturated signal being higher than that of the silver CRE. When comparing the different types of movements, both dry electrodes seem to be less sensitive to horizontal head movement. By contrast, the silver CRE seems to be more sensitive to laughing and deep breathing, which entail rib cage movement.

## 4. Discussion

An electrode for comfortable high spatial resolution surface ECG recording has been manufactured using a textile substrate and with two types of conductive materials, namely silver and PEDOT:PSS. So far, metals such as Ag or AgCl have been used for the construction of these electrodes, but these materials can cause allergies in some individuals. Therefore, the use of totally biocompatible materials, such as some conducting polymers, is a desired alternative. PEDOT:PSS used in this work is a commercial product to which no compound has been added to improve its conductivity or to avoid further problems. However, in view of the results, it would be interesting to add an organic compound such as dimethyl sulfoxide (DMSO) or ethylene glycol to enhance the conductivity one to three orders of magnitude. It should also be noted that some of the manufactured electrodes using PEDOT:PSS had to be discarded since the material had not been deposited uniformly enough to ensure good conductivity and contact area. In this context, some changes to the manufacturing process are required to enhance its reproducibility in terms of correct and uniform deposition of the material. A possible way to improve the response is to first deposit a layer of silver and then on top of this to deposit a layer of PEDOT:PSS [34].

The developed electrodes have been tested in healthy volunteers in order to assess its capability of detecting cardiac activity in patients at rest as the first step toward transferring CREs to clinical applications. It should be noted that in the present work, compared to previous publications [33,34,43], larger CREs were designed. It was taken into account that CREs would be implemented on textile substrates, which could compromise the ability to capture cardiac signals compared to smaller CREs developed on flexible plastic substrates [32,33,39]. Nonetheless, in this regard, it should be noted that the amplitudes of the BC-ECG signals captured with the textile CRE have been higher (more than two times) than those of smaller flexible CREs implemented on plastic substrates [33,35,39]. It has also been verified—as in previous studies—that the amplitudes of the ECG signals captured in position CMV1 are considerably lower than those associated with position CMV2, located on the left side of the patient and closer to the central part of the patient’s heart [33]. Regarding the CREs’ location on the chest for obtaining BC-ECG signals, previous studies carried out by the present research group revealed the superiority of CREs located in chest positions comparable to precordial V1 (CMV1) and precordial V2 (CMV2) for picking up cardiac activity compared to positions comparable to precordial V4R (CMV4R) or comparable to precordial V5 (CMV5) [33]. To simultaneously record cardiac signals in CMV1 and CMV2, an inter-CRE distance of 120 mm was chosen for the CREs’ set design. This distance permits the correct placing of the two CREs in the desired positions on a torso of a medium-build subject. In the case of subjects with a very thin or wide frame, placement of the electrode on the CMV1 position is prioritized. Once the ability of textile CREs to capture cardiac activity has been proven, future work aims to improve the ability of the system to adapt to the patient's anatomy.

Regarding spatial resolution, previous work that used smaller CREs implemented on flexible plastic substrates [33] reported the identification of more ‘local cardiac activity’ as is the case of P1 and P2 atrial waves. Despite the spatial resolution decreasing with increasing size of the CRE [44], the P1 and P2 atrial waves could also be identified in the BC-ECG records performed in the present work with the larger electrodes in this work. These waves are not identifiable in conventional recordings with disc electrodes. At present, there has not yet been a translation of surface high spatial resolution ECG techniques, either with disk electrodes and the application of interpolation techniques or through the use of CRE to clinical practice. In the first case, the use of a large number of monopolar electrodes to carry out the ECG mapping entails the use of complicated recording systems that are not viable in clinical use. The use of CREs could reduce the number of required electrodes. Nonetheless, the best locations to obtain useful diagnostic information should still be studied. Furthermore, the information provided by surface bioelectric signals from CREs has not been analyzed and compared with that of conventional records from standard derivations.

With this work, we aimed to make a prototype that brings the use of CREs closer to clinical use. To do this, systems must still be developed that are comfortable for the patient, are easy to use, and that provide information that is easy for the physician to interpret. In future work, we propose to analyze the behavior of this type of electrode in stress tests and ambulatory recording systems.

## 5. Conclusions

- The use of silver on textiles presents better characteristics than PEDOT:PSS. Even so, techniques that improve the transfer of PEDOT:PSS to textiles could be used.
- Both textile silver and PEDOT:PSS CREs implemented on textile substrates are able to detect surface electrocardiographic activity in standard precordial recording positions, similar to V1 and V2 (CMV1 and CMV2).
- The amplitudes the BC-ECG signals detected with the textile CREs developed are of hundreds of microvolts, which is slightly lower than those of conventional bipolar ECG signals in precordial positions.
- BC-ECGs recorded with silver and PEDOT:PSS textile CREs presented similar signal to noise ratios with these values being similar to those of BC-ECGs from CREs implemented on plastic substrates published in the literature.
- Regarding the saturation and alterations of the BC-ECGs associated with movement of the subject, textile silver CRE showed a more stable response (fewer saturations and alterations) than PEDOT:PSS.
- Regardless the use of silver or PEDOT:PSS, BC-ECG signals captured with the developed textile CREs have a better spatial resolution than that of conventional recordings (lead II). Specifically, BC-ECG signals have an improved capability of recording atrial activity on the surface, with P1 and P2 waves being associated with the activity of the left and right atria identified in most BC-ECG signals.

To sum up, surface ECG records of high spatial resolution could be obtained with a comfortable and simple system using CREs of the proposed dimensions, implemented using screen printing techniques on textile substrates and with Ag ink as the conductive material.

## Figures and Tables

**Table 1:** Concentric ring electrode (CRE) dimensions and distance.

Parameter	Units (mm)
Inner disc diameter	16
Ring internal diameter	36
Ring external diameter	50
Distance (between the discs’ centers)	120

**Table 2:** Inks’ parameters.

Property	Ag C2131014D3	PEDOT:PSS C2100629D1
Solids Content (%)	57.00–59.75	-
Vicosity (Pa.s)	6.5–13.5	0.5–2.0
Curing condition (°C)	130°/3 min	130°/15 min
Sheet resistivity (25 µm)	100 mΩ/sq	500–700 Ω/sq

**Table 3:** Values of equivalent circuit model.

Property	Ag C2131014D3	PEDOT:PSS C2100629D1
R_1_ (Ω)	4.80 × 10^2^	2.73 × 10^3^
R_2_ (Ω)	1.81 × 10^6^	1.85 × 10^6^
Q_2_ (F·s^(α−1)^)	69.79 × 10^−9^	79.96 × 10^−9^
α	0.76	0.79

**Table 4:** Skin-electrode impedance measurements for both external ring and inner disc of the CREs.

	Left Ag	PEDOT:PSS	Right Ag	PEDOT:PSS
External ring impedance (kΩ)	18.3 ± 20.5	27.3 ± 22.3	21.3 ± 22.3	32.0 ± 21.0
Inner disc impedance (kΩ)	25.0 ± 20.1	25.3 ± 24.0	24.0 ± 19.3	32.0 ± 20.1

**Table 5:** Main characteristics of the sensed BC-ECG signal at rest.

	Left		Right	
	Ag	PEDOT:PSS	Ag	PEDOT:PSS
Peak to peak amplitude (μV)	330.2 ± 126.2	363.9 ± 148.0	124.8 ± 133.6	143.8 ± 128.3
SNR (dB)	22.4 ± 6.3	20.1 ± 6.8	20.8 ± 6.6	20.2 ± 3.9

**Table 6:** Mean time percentage in which ECG signal was altered and/or saturated due to intentional movements of the total patients for both electrodes (Ag and PEDOT:PSS) in different positions.

	Left				Right			
	Altered (%)		Saturated (%)		Altered (%)		Saturated (%)	
	Ag	PEDOT:PSS	Ag	PEDOT:PSS	Ag	PEDOT:PSS	Ag	PEDOT:PSS
Head	3.2	10.1	0.0	3.1	5.9	9.6	0.0	1.1
Arm	13.9	28.8	0.9	17.7	13.6	31.6	0.9	12.2
Leg	18.9	36.9	0.1	13.1	24.2	28.1	0.0	10.7
Laughing	18.1	41.4	2.1	26.9	22.9	24.9	4.2	7.1
Breathing	13.9	44.5	6.1	36.1	25.5	29.9	3.5	13.3
μ ± σ	13.6 ± 6.2	32.3 ± 13.8	1.8 ± 2.6	19.4 ± 12.7	18.4 ± 8.4	24.9 ± 8.9	1.7 ± 2.0	8.9 ± 5.0
